# Pathogen Detection and Diagnostic Scenarios in Chronic Prostatitis

**DOI:** 10.3390/diagnostics15060762

**Published:** 2025-03-18

**Authors:** Vittorio Magri, Gianpaolo Perletti, Konstantinos Stamatiou

**Affiliations:** 1 Urology Clinic, ASST Fatebenefratelli Sacco Hospitals, 20026 Milan, Italy; vmdoctor26@gmail.com; 2 Department of Biotechnology and Life Sciences, Section of Medical and Surgical Sciences, University of Insubria, 21100 Varese, Italy; 3 Urology Department, Tzaneio General Hospital, 18536 Piraeus, Greece; stamatiouk@gmail.com

**Keywords:** prostatitis, chronic prostatitis, chronic pelvic pain syndrome, urological infections, sexually transmitted infections

## Abstract

**Background/Objectives:** Chronic prostatitis (CP) is characterized by a variety of symptoms, including pelvic pain, urinary disturbances, and sexual dysfunction, often without clear signs of infection, which complicates its diagnosis. For decades, the NIH consensus definitions and the Meares–Stamey 4-glass test have been the cornerstone of diagnosing and classifying CP. However, emerging research suggests that some cases with negative microbiological findings may still respond to antibacterial therapy, potentially due to undiagnosed infections. This study aimed to compare four lower genito-urinary tract diagnostic methods to identify which is most effective at detecting causative pathogens in CP patients. Two simplified tests, each involving only two specimens, were also simulated. **Methods:** This retrospective study examined a database of patients diagnosed with chronic prostatitis according to NIH criteria. Patients aged 18–59 underwent clinical and microbiological diagnostic assessments using four testing modalities: the Meares–Stamey 4-glass “gold standard” test, the two-glass pre–post-massage test, and two tests incorporating post-massage semen samples, namely the five-glass test and the VB2-semen test. The diagnostic outcomes and pathogen detection rates for each test were compared using the ANOVA and the Pearson’s chi-squared tests. **Results:** Compared to the four-glass and two-glass tests, the five-glass and VB2-semen tests detected similar proportions of *E. coli* and other Gram-negative traditional prostatic pathogens. However, they were more effective in detecting significantly higher percentages of Enterococci. Moreover, the five-glass and VB2-semen tests, which included semen samples, identified a broader spectrum of pathogens and significantly higher proportions of sexually transmitted pathogens. **Conclusions:** Tests that included semen samples were more effective at detecting Gram-positive pathogens such as Enterococci and sexually transmitted pathogens. We advocate for incorporating semen samples into the standard four-glass test to enhance diagnostic accuracy and improve the targeted antibacterial treatment of chronic prostatitis.

## 1. Introduction

Chronic prostatitis encompasses a range of syndromes characterized by symptoms affecting the prostate and resulting from various potential etiological agents. Significant advancements in the understanding and classification of these conditions have been made over the past 30 years, alongside the creation of improved tools for assessing symptom severity, such as the NIH-Chronic Prostatitis Symptom Index (NIH-CPSI) [1]. The term chronic prostatitis (CP) includes two distinct conditions, which are classified according to NIH criteria into two primary categories: Category II, chronic bacterial prostatitis (CBP), and Category III, chronic prostatitis/chronic pelvic pain syndrome (CP/CPPS) [2]. Both categories share common symptoms, including persistent pelvic pain, lower abdominal or perineal discomfort, and ejaculatory pain. Other common symptoms include urinary issues, such as frequent and/or difficult urination, urgency, dysuria, and nocturia. Sexual dysfunction, including erectile difficulties, may also be present, along with anxiety or depression. Symptoms often fluctuate in intensity and can persist for months or even years, significantly affecting quality of life. Although some patients may experience recurrent infections or flare-ups of prostatitis, CP does not always present with signs of infection, complicating the diagnostic process.

One of the most notable developments in recent research has been the introduction of the UPOINT framework. UPOINT is a diagnostic and therapeutic algorithm for CP/CPPS that focuses on six domains: urinary symptoms, psychosocial factors, organ-specific factors (e.g., prostate issues), infection, neurological factors, and tenderness (musculoskeletal pain, pelvic floor dysfunction) [3]. A category for sexual dysfunction has been shown to complement the UPOINT domains, enhancing correlations between domains and symptoms [4]. By evaluating each domain, UPOINT(S) allows clinicians to identify the underlying contributors to symptoms and tailor multimodal treatments, such as medications, physical therapy, or psychological interventions, offering a comprehensive and personalized approach to managing CP [5]. Among these interventions, antibacterial therapy is central to the treatment of Category II CBP and is often administered empirically to newly diagnosed CP/CPPS cases.

The Meares–Stamey 4-glass test, introduced in 1968, remains the gold standard for diagnosing CBP and for the differential diagnosis of CP/CPPS. The test involves the collection of four urine samples: a first-stream urine sample, a midstream urine sample, prostate secretions expressed during a prostatic massage, and a post-massage urine sample [6]. By comparing the bacterial growth and white blood cell counts in each sample, the test can confirm a prostate-localized infection. Three decades after the development of the 4-glass test, a two-glass pre- and post-massage test (PPMT) was proposed as an alternative, reducing the number of samples to pre- and post-massage urine [7]. Despite the introduction of the simpler two-glass test, some specialists have further limited their microbiological analysis to only midstream urine or ejaculate specimens [8].

While CP/CPPS has historically been considered a non-bacterial condition, increasing evidence suggests that some cases may respond to antibacterial therapy. This implies that low-grade undiagnosed bacterial infections or microbial imbalances could contribute to the persistence of symptoms [9,10]. This raises the question: does the response of some symptomatic CP/CPPS patients to antibacterial therapy indicate an occult infection of the prostate? To investigate this hypothesis, research groups, including ours, have explored whether adding semen cultures to the Meares–Stamey 4-glass test could help detect occult infections in CP/CPPS cases previously thought to be non-bacterial [11]. The authors of these studies proposed that such a “five-glass test” might reveal chronic or persistent infections that are difficult to detect using conventional segmented tests.

Our Mediterranean Prostatitis Research Group has compiled a database of 1047 microbiological tests performed using the traditional Meares–Stamey 4-glass test or an enhanced version of the five-glass protocol. We analyzed this database to compare the diagnostic outcomes of the five-glass, four-glass, and two variants of the two-glass tests. The goal of this investigation was to assess which segmented urinary tract test efficiently isolates and detects the widest range and highest number of causative or co-causative pathogens in symptomatic patients affected by CBP or CP/CPPS.

## 2. Materials and Methods

This observational, non-interventional study was based on a retrospective analysis of a database that included patients who underwent standard diagnostic and therapeutic protocols at a secondary care urology center. The database was compiled between January 2004 and December 2024. All patients provided written consent for the processing and anonymous publication of their clinical data. This study was approved by the institution’s ethics committee.

### 2.1. Inclusion Criteria

Patients aged 18 to 59 years were included in the database if they met the NIH criteria for chronic prostatitis (NIDDK Chronic Prostatitis Workshop, Bethesda, MD, USA, 7–8 December 1995) [2] and had a positive microbiological result from a segmented test of the lower urinary tract. Patients were excluded if they met any of the following criteria: Category I acute bacterial prostatitis, urethral discharge, antibiotic therapy or medications affecting the prostate within the 3 months prior to their chronic prostatitis diagnosis, indwelling catheters, cystostomy, ureterostomy, previous prostate surgery, or a diagnosis of prostate cancer.

### 2.2. Clinical Assessments

The severity of CP symptoms was assessed using the NIH Chronic Prostatitis Symptom Index (NIH-CPSI) and the International Prostate Symptom Score (IPSS). Additional clinical evaluations included serum prostate-specific antigen (PSA) levels, the urine peak flow rate (Qmax), and post-micturition residual urine volume in the bladder. Urological assessments also included a digital rectal examination of the prostate. Diagnostic imaging of the prostate, bladder, and seminal vesicles was performed for each patient using both trans-abdominal and trans-rectal ultrasonography.

### 2.3. Microbiological Assessments

Patients underwent microbiological tests following the standard clinical practices of the hosting institution. All patients underwent the Meares–Stamey 4-glass segmented localization test [6]. This test could be followed by a prostatic massage and the collection of a semen specimen, depending on the specialist’s decision. Prior to semen collection, patients were instructed to cleanse their hands and glans penis with antiseptic soap.

**Figure 1:**
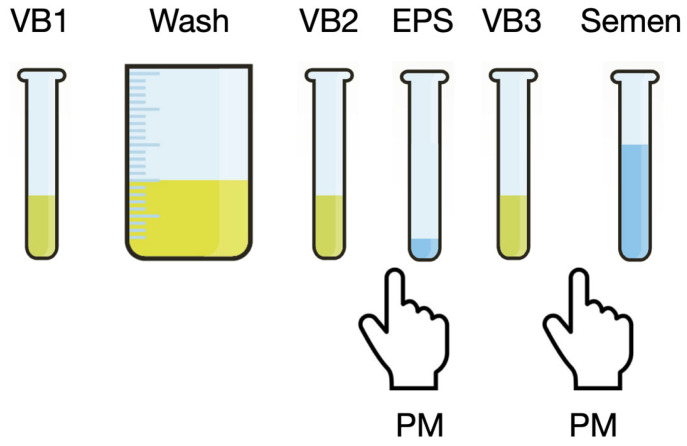
The 5-glass test. Four urine samples were collected: a first-stream urine sample (VB1, ~15 mL), was followed by a washing step during which ~150–200 mL of urine was voided. A midstream urine sample (VB2, ~15 mL) was subsequently collected, followed by prostatic massage (PM) and the collection of prostate secretions expressed through massage (EPS). A post-massage urine sample (VB3, ~15 mL) was then collected. Post-massage semen was collected afterwards by masturbation.

The standard 4-glass lower urinary tract segmented test, according to Meares and Stamey, involves the collection of a first-void urine sample (VB1, ~15 mL), followed by a urethral washing step, during which 150–200 mL was collected and discarded in our methodology. This was followed by the collection of a midstream urine sample (VB2, ~15 mL). Prostate secretions were then obtained during a prostatic massage (EPS), and then a post-massage urine sample was collected (VB3, ~15 mL) [6]. The 5-glass test consisted of the complete 4-glass test, followed by the collection of a post-massage semen sample, as described in Figure 1.

For the simulation of microbiological analyses involving only two specimens (or “glasses”), such as the PPMT and VB2-semen test, the results from 4-glass and 5-glass tests were used. In the case of the PPMT, the results from the VB1 and EPS specimens, which were part of the 4-glass test, were hidden from the data analyzer. Similarly, for the VB2-semen test, the microbiological results from the VB1, EPS, and VB3 specimens obtained during the complete 5-glass test were hidden in the database.

Samples were analyzed for the presence and load of traditional prostatic uropathogens, including *Escherichia coli*, other *Enterobacteriaceae*, *Pseudomonas aeruginosa*, *Enterococcus* spp., and *Staphylococcus aureus*, as well as other aerobic and anaerobic pathogens. This included sexually transmitted bacteria, protozoa, and fungi, such as *Neisseria gonorrhoeae*, various *Mycoplasmata*, *Trichomonas vaginalis,* and *Candida* spp.

The bacterial load cutoff for a positive specimen was set at 10^3^ colony-forming units/mL, based on the diagnostic criteria established by Naber and the Lomefloxacin Prostatitis Study Group [12] and the thresholds used by other research groups investigating microbiological analyses of semen in prostatitis, e.g., those in [13,14].

### 2.4. Statistical Analysis

Differences between normally distributed variables assessed across multiple groups were analyzed using a one-way analysis of variance (ANOVA), followed by Tukey’s post hoc Honestly Significant Difference (HSD) test to evaluate the significance of pairwise comparisons. The significance of differences between two or more proportions or frequencies was assessed using Pearson’s chi-squared test (χ^2^) for sample sizes greater than 5, or Fisher’s exact test for smaller sample sizes. All statistical analyses were conducted using the *R* environment for data analysis and statistical computation.

## 3. Results

The database analysis identified 1047 patients (mean age range: 45.4–47.3 years) who exhibited signs and symptoms of chronic prostatitis and met the inclusion criteria for this retrospective study. Of these, 797 patients underwent the five-glass test, and 250 patients underwent the four-glass test.

### 3.1. Baseline Clinical Assessments

Baseline clinical data and statistics are summarized in Table 1. The participants’ serum PSA concentration ranged from 2.35 to 2.67 ng/mL, with no significant differences detected through the ANOVA test. Similarly, no significant differences were observed in peak urinary flow (range: 16.07–16.22 mL/s) or the post-void residual volume of urine retained in the bladder (range: 38.73–45.04 mL).

All the components of the NIH-CPSI were analyzed, including the pain symptom score, voiding disturbances score, quality of life (QoL) impact score, and the total score across these domains. The pain symptom score (range: 21.83–22.56), QoL impact score (range: 7.48–7.69), and total CPSI score (range: 21.83–22.56) showed no significant differences between groups or in pairwise comparisons (*p* > 0.05 in all cases, ANOVA and Tukey’s post hoc test, Table 1).

Voiding symptom scores showed no significant differences when the five-glass test and four-glass test were compared with each other, or with the two-glass test or the VB2-semen test. However, an exception was observed in the comparison of voiding symptom scores between the groups that underwent the two-glass pre- and post-massage test and the VB2-semen test. In this case, the two-glass test was associated with slightly but significantly higher voiding symptoms. This was reflected in significantly higher voiding scores on the NIH-CPSI (scores: 5.05 vs. 4.43, *p* = 0.008, Tukey’s test) and higher scores on the IPSS test, which primarily assesses voiding symptoms (scores: 13.56 vs. 11.45, *p* = 0.0002, Tukey’s test).

In general, our data analysis indicated that the primary signs and symptoms of prostatitis did not significantly differ among patients diagnosed with the four different microbiological tests. With the exception of the observed differences in voiding disturbances, patients undergoing microbiological assessments appeared to present with comparable pain symptom severity and with similar impacts of the disease on their QoL.

### 3.2. General Microbiological Assessments

A total of 1047 patients with documented signs and symptoms of CP underwent lower urinary tract segmented testing. Table 2 shows the percentage of pathogens detected, stratified by different bacterial load cutoffs (10^3^, 10^4^, 10^5^, 10^6^ cfu/mL).

Table 3 outlines the species, absolute numbers, and frequencies of pathogens detected across the four microbiological tests, along with the results of the statistical analysis. Pathogens were categorized into three main groups: traditional prostatic uropathogens, sexually transmitted pathogens, and other pathogens.

A total of 1150 pathogens were identified. One-hundred and three patients (9.8%) were infected by two different species. The four-glass and two-glass tests detected 278 and 165 pathogens, respectively, generating a 40% difference. The five-glass and VB2-semen tests detected 872 and 794 pathogens, respectively, a 9% difference. The five-glass and VB2-semen tests detected significantly higher proportions of the total pathogens compared to the four-glass and two-glass tests, when calculated as a percentage of the total number of tests performed.

In the five-glass test, 54% of pathogens were isolated exclusively from the semen specimen, 26% were only detected in the EPS and/or VB3 samples, and 20% of pathogens were isolated from both semen and EPS/VB3 samples.

### 3.3. Traditional Uropathogens

The four-glass test detected 1.7 times the number of traditional prostatic pathogens compared to the two-glass test (*n* = 196 vs. 115, a 41% difference). The five-glass test detected 1.2 times the number of traditional uropathogens compared to the VB2-semen test (*n* = 543 vs. 453, a 16% difference). The most common pathogens identified were *Escherichia coli* and *Enterococcus faecalis*.

All tests revealed similar proportions of traditional uropathogens, with no statistically significant differences, when calculated as a percentage of the total pathogens detected. However, compared to the four-glass and two-glass tests, tests incorporating a semen sample (five-glass and VB2-semen) consistently detected significantly higher proportions of Gram-positive species (five-glass: 35%; four-glass: 24%; two-glass: 19%; VB2-semen: 43%) when calculated as a percentage of the total number of traditional uropathogens (Table 3). No significant differences were observed in the detection frequency of Gram-negative uropathogenic species (such as *E. coli*, other *Enterobacteriaceae*, and *Pseudomonas aeruginosa*) between the five-glass, four-glass, and two-glass tests (five-glass: 65%; four-glass: 76%; two-glass: 81%). However, the VB2-semen test detected a lower proportion of Gram-negative species (57%) compared to the four-glass and two-glass tests. This difference can be explained by the higher percentages of Gram-positive species detected by the former test, which in turn reduces the fraction of Gram-negative species isolated.

#### 3.3.1. Enterobacteriaceae

The four-glass test detected 1.7 times more *E. coli* compared to the two-glass test (*n* = 123 vs. 71, Table 3). The five-glass test detected 1.6 times more *E. coli* compared to the VB2-semen test (*n* = 251 vs. 159). When comparing the percentages of *E. coli* among traditional uropathogens, the four-glass test detected a significantly higher proportion of this pathogen compared to the five-glass and VB2-semen tests. The lower proportion of *E. coli* detected by the five-glass and VB2-semen tests can be explained by the higher percentages of other uropathogenic species detected by these tests. This increases the overall number of species isolated, thus decreasing the relative percentage of *E. coli*.

The five-glass test detected a significantly higher percentage of *E. coli* compared to the VB2-semen test, whereas the latter detected significantly higher proportions of *Klebsiella* spp. compared to the four-glass test.

#### 3.3.2. Enterococci

The four-glass test detected 1.9 times more Enterococci compared to the two-glass test (*n* = 37 vs. 19). The VB2-semen test detected similar amounts of Enterococci compared to the five-glass test (*n* = 182 vs. 172, a 1.06-fold difference, Table 3). The five-glass test detected significantly higher proportions of Enterococci (*E. faecalis* and *E. faecium*) compared to the four-glass and two-glass tests. Additionally, the VB2-semen test detected significantly higher proportions of Enterococci compared to the five-glass, four-glass, and two-glass tests. This suggests that tests incorporating semen samples may enhance the detection rate of these Gram-positive prostatic pathogens.

### 3.4. Sexually Transmitted Pathogens

The five-glass, four-glass, two-glass, and VB2-semen tests detected a total of 175, 38, 22, and 174 sexually transmitted (ST) pathogens, respectively (Table 3). The most common ST pathogens were *Ureaplasma urealyticum* and *Chlamydia trachomatis*. Tests incorporating semen samples detected a broader range of ST species, including *Neisseria gonorrhoeae*, *Candida* spp., and *Trichomonas vaginalis*.

The five-glass and VB2-semen tests identified significantly higher proportions of ST pathogens compared to the two-glass test. In terms of ST pathogens, the four-glass test detected a significantly higher proportion of *C. trachomatis* compared to the VB2-semen test. This difference can be attributed to the broader diversity and higher percentage of other species detected by the VB2-semen test, which increases the total number of ST pathogens detected and lowers, in turn, their relative percentages.

### 3.5. Other Pathogens

The five-glass, four-glass, two-glass, and VB2-semen tests detected a total of 154, 44, 28, and 167 other pathogens, respectively (Table 3). The most commonly detected species included Group B *Streptococcus beta hemolyticus*, *Haemophilus parainfluenzae*, and coagulase-negative Staphylococci. All tests yielded similar proportions of pathogens, except for the four-glass test, which detected significantly higher percentages of *Streptococcus agalactiae* compared to the five-glass and VB2-semen tests.

## 4. Discussion

**Figure 2:**
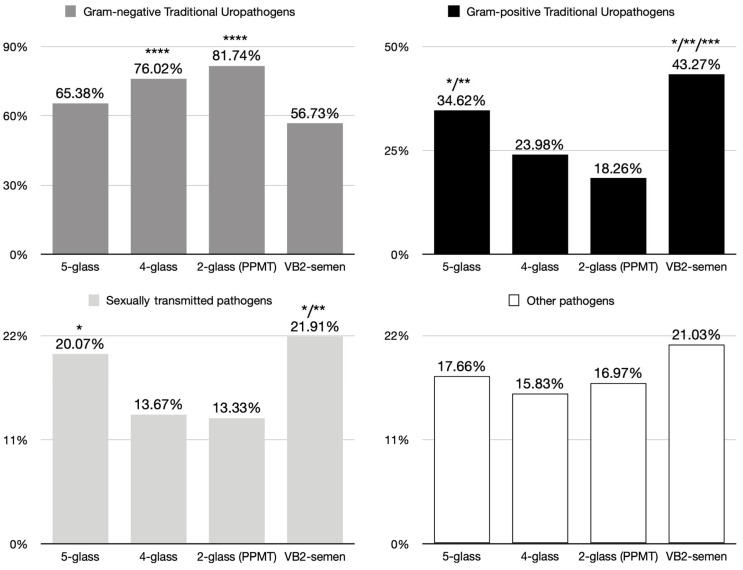
Comparative summary showing the proportions of Gram-positive and Gram-negative traditional prostatic uropathogens, of sexually transmitted pathogens, and of other pathogens isolated with the 5-glass, 4-glass, 2-glass PPMT, and VB2-semen tests. Raw data and statistics are presented in Table 3. (*), *p* < 0.05 vs. 4-glass test; (**), *p* < 0.05 vs. 2-glass test; (***), *p* < 0.05 vs. 5-glass test; (****), *p* < 0.05 vs. VB2-semen test, using Pearson’s Chi-square analysis.

The present study compared the outcomes of the traditional four-glass test, as described by Meares and Stamey, performed in a group cohort of 250 symptomatic patients, with the five-glass test, which included a post-massage semen sample collected at the end of the procedure, performed in 797 patients. Additionally, two simplified tests were simulated by hiding the VB1, VB3, or EPS samples, generating a pre- and post-massage two-glass test and a VB2-semen test based solely on the VB2-VB3 and VB2-semen specimens. The results from both the five-glass and four-glass tests were similar in their ability to detect Gram-negative prostatic pathogens and various non-traditional uropathogenic species, categorized as “other pathogens” (Figure 2). This group includes, for example, coryneforms, whose role in bacterial prostatitis, particularly in patients with severe leukocytospermia, has been supported by multiple studies [15].

Notably, Table 2 shows that 30% to 43% of symptomatic patients diagnosed with an infection based on the 10^3^ cfu cutoff would have remained undiagnosed if higher thresholds for positivity—adopted in some hospital settings—had been used.

A noteworthy finding of this study was the superior ability of the five-glass and VB2-semen tests to detect Gram-positive uropathogenic species, particularly Enterococci (Figure 2). Enterococci accounted for 32% and 40% of all traditional uropathogens detected, respectively, compared to the 19% detected in the four-glass test and the 17% in the two-glass test. Since the latter tests do not include the collection and microbiological analysis of semen specimens, the inclusion of semen may add diagnostic value to the traditional four-glass test, enhancing the detection of Gram-positive etiological agents of infectious prostatitis.

In contrast to Gram-negative *Enterobacteriaceae*, Enterococci are notoriously difficult to eradicate. These organisms are naturally resistant to cephalosporins, aminoglycosides, and quinupristin-dalfopristin and almost universally resistant to vancomycin. Notably, the ability of Enterococci to form tightly encapsulated biofilms in calcified regions of the prostate gland may explain their resistance to both detection and eradication [16,17]. In this context, the mechanical action of ejaculation, preceded by a prostatic massage, may facilitate the detachment of biofilm-embedded Enterococci, thereby aiding in their recovery in semen samples.

In the Mediterranean region, Enterococci account for approximately 21% of prostatitis-causing pathogens. The growing prevalence of multidrug-resistant Enterococci, particularly in Southern Europe, Northern Africa, and the Middle East, represents a significant public health concern that demands urgent attention and optimal diagnostic and therapeutic strategies [18].

Traditionally, *E. coli* has been considered the major etiological agent of bacterial prostatitis. However, as early as 2011, we and others observed a sharp increase in cases of enterococcal prostatitis in Europe [19]. This increase, initially observed in anecdotal reports among specialists, has since been confirmed by longitudinal studies [20]. In light of this, the accurate detection of bacterial infections in the prostate caused by Enterococci is of primary importance for both patient care and public health.

An additional interesting finding of this study was the significantly enhanced detection of sexually transmitted pathogens by the microbiological assays which included the collection and analysis of semen samples (Figure 2). Notably, when the five-glass and four-glass tests were compared, the detection of sexually transmitted pathogens in semen occurred in the absence of evidence of urethral infection in the first-void urine sample (VB1). Furthermore, semen samples were collected after repeated urethral washing steps prior to the collection of the patient’s ejaculate, as shown in Figure 1. Over the past 20 years, in our hospital facilities, the techniques used for detecting certain sexually transmitted pathogens have evolved. For example, *C. trachomatis* was initially detected by non-PCR methods, such as cell culture and enzymatic essays, until the late 2000s, at which point PCR became the standard. The switch to PCR-based techniques likely increased the sensitivity of our detection of these pathogens. However, given that the four-glass and five-glass tests have been conducted in parallel over the years, any potential detection bias introduced by differing pathogen detection methods may have been attenuated by the longitudinal design of the present study.

The question of whether sexually transmitted pathogens are etiological determinants of chronic prostatitis remains unresolved. However, this hypothesis is supported by several studies, particularly those focusing on *Chlamydia trachomatis* and various *Mycoplasmata* [21,22,23,24]. In addition, DNA from both *Trichomonas vaginalis* and *Neisseria gonorrhoeae* has been consistently isolated from the semen samples of patients with prostatitis symptoms [25]. It is well established that a bacterial infection of the prostate can result from ascending urethral infection or the reflux of infected urine into the prostatic ducts draining into the posterior urethra. Moreover, bacterial prostatitis and urinary/genital tract infections, including sexually transmitted infections, share several similarities, including host responses, which lead to increased loads of polymorphonuclear leukocytes and macrophages in prostatic secretions.

In the original definition of chronic prostatitis categories, chronic bacterial prostatitis was considered an infectious disease, whereas chronic prostatitis/chronic pelvic pain syndrome could be diagnosed in the absence of microbiological evidence of causative pathogens [2]. Thus, CP/CPPS partially or completely replaced a condition previously referred to as abacterial prostatodynia. In the past decade, the distinction between bacterial and non-bacterial forms of chronic prostatitis has become increasingly blurred. This paradigm shift occurred with the introduction of the UPOINT diagnostic and therapeutic algorithm, which includes an “infection” domain [3,4], suggesting that an infectious etiological component may exist in cases of CP/CPPS previously defined as non-bacterial, idiopathic, or even psychogenic. In this regard, studies examining the semen microbiome of CP patients have shown a significant dysregulation of the prostate microbiota, with a potential prevalence of anaerobic species in some cases [26,27,28].

Consequently, the presence of an infectious component is now recognized as a key feature in variants of the disease previously regarded as “non-bacterial”. Furthermore, meta-analyses have shown that antibiotic therapy can lead to the partial or complete resolution of the complex symptoms of CP/CPPS, with significant reductions in pain and voiding symptom scores, as assessed by the NIH-CPSI test, compared to placebo [10], though this finding has been questioned by some experts [29]. This has prompted researchers to suggest that antibacterial therapy may be considered for newly diagnosed, antimicrobial-naive patients (level of evidence 1: grade of recommendation C-D), as CP/CPPS may be caused by uncultured or unculturable organisms in some patients [30,31].

The subjective experience of specialist caregivers plays a crucial role in the outcomes of prostatitis diagnostic tests. An interesting study highlighted the considerable variability in the rate of positive four-glass tests across different urology centers. Centers with a dedicated prostatitis specialist had a significantly higher rate of positive diagnoses (64.3%) compared to general urological departments (31.4%) [32]. In this context, the proper execution of a prostatic massage, directed from the distal portion of the gland toward the medial prostatic ducts, is crucial for obtaining sufficient amounts of prostatic secretions. Furthermore, we hypothesize that adding a semen sample could reduce the subjective variation in diagnostic performance related to sample collection and massage. While the complexity of the five-glass test may discourage specialists from performing it, one might wonder whether a simplified diagnostic test, involving one or two specimens, could still yield reliable results, given the considerable variability in diagnostic efficiency. Therefore, despite their complexity, tests including semen samples may uncover infections that would otherwise remain undetected and undiagnosed.

The diagnostic sensitivity of segmented tests, including the five-glass test, can be further enhanced by utilizing molecular diagnostic methods. The polymerase chain reaction (PCR) of specific bacterial sequences, such as the 16S ribosomal RNA (rRNA), and other nucleic acid-based technologies are among the most promising techniques [33]. Several studies have been conducted in CP patients, yielding interesting results. For example, Xiao and colleagues detected atypical pathogens in the expressed prostatic secretion (EPS) from CP/CPPS patients using the PCR, cloning, and sequencing of the entire 16S rDNA product. Sexually transmitted pathogens such as *Ureaplasma urealyticum* and *Chlamydia trachomatis* were identified, with bacterial loads significantly correlated with symptom severity [34]. The study of the prostate microbiome is gaining momentum. Alterations in microbiome composition and dysbiosis are believed to be among the causative agents of CP/CPPS and/or CBP, as demonstrated in a number of studies and meta-analyses summarized in [35,36,37,38]. Most studies in this field have utilized 16S rRNA amplification and sequencing techniques.

It is worth noting that in this study, patients diagnosed with bacterial infections through the five-glass or four-glass tests exhibited similar clinical signs and symptoms, with no statistically significant differences in severity. This finding essentially excludes the possibility that semen samples could harbor pathogens unrelated to the clinical presentation of chronic prostatitis. If the semen contained incidental contaminants, one might expect to see milder symptoms—particularly pain—in a substantial proportion of patients undergoing the five-glass or VB2-semen tests.

This observation is also supported by the results of a previous study performed by members of our research group on a cohort of 696 symptomatic patients [11]. Patients subjected to a four-glass, a two-glass, or a different version of the five-glass test exhibited baseline NIH-CPSI symptom scores that were not significantly different among the groups. Interestingly, antibacterial treatment led to a marked decrease in symptom scores across all groups, with no significant intergroup differences. Symptom scores remained stable and non-significantly different among groups. Moreover, disease remission persisted over a period of 24 months. A longitudinal 2-year study is currently ongoing in our hospital facility.

Over the past two decades, several research groups have examined the feasibility and reliability of alternative segmented tests which have been designed to include semen specimens. For example, Wang and Wang proposed a five-glass test that included the collection of VB1, VB2, EPS, VB3, and semen. In a study of 200 symptomatic patients, they reported a 100% infection rate, with Gram-positive species being the most frequently isolated pathogens. However, the authors argued that semen collection might recover flora from various genital regions, including the deferens tract [39]. In this regard, it is important to note that semen is a composite fluid primary composed of secretions from the seminal vesicles and the prostate, which contribute 65–75% and 20–30% of its volume, respectively. Thus, the species isolated from semen may reflect bacterial colonization extending to these anatomical areas.

An interesting 2018 study by Iovene and colleagues demonstrated that enriched cultures from semen specimens effectively detected pathogens in 73% of a cohort of 120 chronic prostatitis patients, with 100% sensitivity. Interestingly, biofilm-producing Enterococci were identified as the most common pathogens [13]. A comprehensive 2016 study in Spain, involving 761 symptomatic CP patients who underwent a segmented test involving the collection of semen and post-ejaculate urine, found that 96% of patients had positive semen cultures, while 22.9% had positive post-ejaculate urine cultures. The sensitivity, specificity, positive predictive value, and negative predictive value of semen were 96.7%, 95.9%, 84.3%, and 99.3%, respectively. Again, *Enterococcus faecalis* was the primary pathogen identified (37.7%), a value closely aligning with the findings of the present study (32% and 40% in the five-glass and VB2-semen tests, respectively) [40]. The increased detection of *Enterococcus faecalis* has also been confirmed in a 2023 systematic review on the etiology and antibiotic resistance profile of CBP by Mendoza-Rodriguez and coworkers, who found this pathogen in approximately 47% of CP cases [41]. In 2006, Budìa and colleagues reported similar sensitivity values for semen cultures, with 100% sensitivity for Gram-positive and 97% for Gram-negative pathogens, compared to 82.4% and 16.1% for EPS, respectively [42]. Zegarra-Montes et al. compared a semen-only test with the four-glass test and found that semen culture had a sensitivity of 45% and a specificity of 94%. They concluded that while a negative semen culture does not rule out CBP, a positive test in a patient with a high pre-test probability of CBP may be sufficient to initiate antibiotic treatment. The authors recommended that the Meares–Stamey 4-glass test remain the reference standard for diagnosing CBP in clinical practice [43].

The increased detection of causative pathogens of CBP or CP/CPPS by means of a more complex test, such as the five-glass test, may have significant therapeutic and clinical practice implications. As previously mentioned in this article, empirical therapy is frequently administered to newly diagnosed cases of CP/CPPS. However, detecting pathogens that would be undetected by a simplified two-glass test or a single-sample test may reduce the likelihood of resorting to empirical therapy, which may end up being off-targeted or ineffective, particularly in the context of rising pathogen resistance.

## 5. Conclusions

In conclusion, our study highlights the value of semen analysis as a valuable addition to the Meares–Stamey 4-glass test, although it should not be considered a substitute. Most studies reviewed emphasize that Gram-positive species, especially Enterococci, are frequently isolated from semen samples. As such, incorporating a semen analysis could enhance the specificity of the four-glass test in identifying these critical prostate pathogens. Our findings also indicate that simplified tests, such as the two-glass and VB2-semen tests, show considerable deviations from the results of the more comprehensive four-glass and five-glass segmented tests. In our view, simplified tests—or those based on a single specimen (like post-massage urine or semen)—carry a higher risk of leading to inaccurate diagnostic conclusions in symptomatic individuals.

While the five-glass test is undeniably more complex, costly, and time-consuming, these drawbacks should not overshadow its benefits. We believe that, in cases where a simplified test like the PPMT yields negative results in moderately or severely symptomatic patients, re-testing with the five-glass test can help identify an underlying pathogen, thus improving the chances of curing an undiagnosed infection.

Thus, despite its non-absolute specificity and its increased complexity, the ability of the five-glass test to detect higher proportions of important prostatic pathogens may significantly benefit patients suffering from infections that profoundly impact their quality of life and psychosocial well-being.

## Figures and Tables

**Table 1:** Baseline clinical data of a cohort of 1047 chronic prostatitis patients subjected to microbiological assessments conducted according to four different methods.

Test	5-Glass	4-Glass	2-Glass	VB2-Semen	One-Way ANOVA	Tukey’s HSD Post Hoc Test
Mean age (±SD)	46.51 ± 13.42	47.34 ± 14.55	47.42 ± 15.01	45.39 ± 12.96	*p* = 0.06	*p* > 0.05, all comparisons
Mean PSA, ng/mL (±SD)	2.50 ± 3.068	2.67 ± 3.47	2.58 ± 2.87	2.35 ± 2.75	*p* = 0.42	*p* > 0.05, all comparisons
Mean Qmax, mL/S (±SD)	16.19 ± 9.41	16.22 ± 10.15	16.09 ± 10.41	16.07 ± 8.67	*p* = 0.99	*p* > 0.05, all comparisons
Post-void residual bladder urine, mL	40.05 ± 55.11	42.38 ± 58.31	45.04 ± 59.37	38.73 ± 52.72	*p* = 0.42	*p* > 0.05, all comparisons
NIH-CPSI total score (mean ± SD)	21.83 ± 7.19	22.14 ± 6.98	22.56 ± 7.12	21.98 ± 7.27	*p* = 0.55	*p* > 0.05, all comparisons
NIH pain score (mean ± SD)	10.12 ± 5.48	10.13 ± 7.62	10.73 ± 9.59	10.10 ± 6.81	*p* = 0.61	*p* > 0.05, all comparisons
NIH voiding symptom score (mean ± SD)	4.61 ± 2.68	4.89 ± 2.61	5.05 ± 2.57	4.43 ± 2.71	***p* = 0.047**	*p* > 0.05, for all comparisons, except: 2-glass vs. VB2-semen, ***p* = 0.0081**
NIH QoL impact score (mean ± SD)	7.48 ± 2.71	7.68 ± 2.70	7.69 ± 2.63	7.60 ± 2.71	*p* = 0.58	*p* > 0.05, all comparisons
IPSS (mean ± SD)	11.93 ± 6.85	12.56 ± 6.81	13.56 ± 7.04	11.45 ± 7.02	***p* = 0.0003**	*p* > 0.05, for all comparisons, except: 2-glass vs. VB2-semen, ***p* = 0.0002**

Qmax, peak urine flow; NIH-CPSI, NIH Chronic Prostatitis Symptom Index; QoL, quality of life; IPSS, International Prostate Symptom Score; HSD, Tukey’s “Honestly Significant Difference”; SD, Standard Deviation. Statistically significant data are shown in bold.

**Table 2:** Percentage of pathogens detected in expressed prostatic secretions (EPSs), post-massage urine (VB3), and semen, stratified according to different bacterial load cutoffs.

Pathogen Load, cfu/mL	EPS, %	VB3, %	Semen, %
10^3^	43.62	32.58	30.29
10^4^	29.53	17.98	23.72
10^5^	20.81	26.22	30.93
10^6^	6.04	23.22	15.06
Total	100	100	100

**Table 3:** Pathogens detected using four different lower urinary tract segmented microbiological tests in a cohort of 1047 patients showing signs and symptoms of chronic prostatitis. Statistically significant data are shown in bold.

	Diagnostic Test								Significance of Difference Between Diagnostic Tests (Pearson’s *χ*^2^ or Fisher’s Exact Test)					
	**5-Glass**		**4-Glass**		**2-Glass**		**VB2-S**		**5G vs. 4G**	**5G vs. 2G**	**5G vs. VB2-S**	**4G vs. 2G**	**4G vs. VB2-S**	**2G vs. VB2-S**
**Species**	***n***	**%**	***n***	**%**	***n***	**%**	***n***	**%**	***p***	***p***	***p***	***p***	***p***	***p***
*Escherichia coli*	251	46.22	123	62.76	71	61.74	159	35.10	**0.034**	0.098	**0.033**	0.94	**0.0002**	**0.0023**
*Enterococcus faecalis*	172	31.68	37	18.88	19	16.52	182	40.18	**0.007**	**0.011**	**0.038**	0.67	**<0.0001**	**0.0003**
*Proteus Myrabilis*	28	5.16	12	6.12	8	6.96	23	5.08	0.65	0.48	0.98	0.78	0.67	0.49
*Klebsiella* spp.	22	4.05	3	1.53	3	2.61	22	4.86	0.11	0.59	0.51	0.67	**0.046**	0.44
*Morganella Morganii*	22	4.05	6	3.06	5	4.35	19	4.19	0.52	0.80	0.86	0.54	0.46	0.99
*Pseudomonas aeruginosa*	17	3.13	3	1.53	5	4.35	12	2.65	0.31	0.56	0.69	0.15	0.57	0.37
*Staphylococcus aureus*	14	2.58	9	4.59	2	1.74	12	2.65	0.19	0.99	0.91	0.34	0.24	0.74
*Citrobacter Koseri*	10	1.84	1	0.51	1	0.87	17	3.75	0.30	0.99	0.23	0.99	0.21	0.71
*Enterobacter cloacae*	5	0.92	1	0.51	1	0.87	5	1.10	0.99	0.99	0.76	0.99	0.67	0.99
*Enterococcus faecium*	2	0.37	1	0.51	0	0.00	2	0.44	0.99	n.a.	0.99	n.a.	0.99	n.a.
**Total Gram-negative**	**355**	**65.38**	**149**	**76.02**	**94**	**81.74**	**257**	**56.73**	0.23	0.15	0.17	0.68	**0.028**	**0.021**
**Total Gram-positive**	**188**	**34.62**	**47**	**23.98**	**21**	**18.26**	**196**	**43.27**	**0.037**	**0.009**	**0.044**	0.34	**0.0006**	**0.0003**
**Total traditional uropathogens**	**543**	**100**	**196**	**100**	**115**	**100**	**453**	**100**						
**Traditional uropathogens/total tested population (*n* = 1047),%**	**51.86**		**18.72**		**10.98**		**43.27**							
*Ureaplasma urealyticum*	118	67.43	21	55.26	12	54.55	124	71.26	0.51	0.57	0.74	0.97	0.39	0.47
*Chlamydia trachomatis*	40	22.86	16	42.11	7	31.82	36	20.69	0.074	0.47	0.69	0.59	**0.039**	0.35
*Mycoplasma hominis*	10	5.71	0	0.00	3	13.64	9	5.17	n.a.	0.19	0.83	n.a.	n.a.	0.16
*Neisseria gonorrheae*	4	2.29	1	2.63	0	0.00	2	1.15	0.99	n.a.	0.68	n.a.	0.45	n.a.
*Candida* spp.	2	1.14	0	0.00	0	0.00	2	1.15	n.a.	n.a.	0.99	n.a.	n.a.	n.a.
*Trichomonas Vaginalis*	1	0.57	0	0.00	0	0.00	1	0.57	n.a.	n.a.	0.99	n.a.	n.a.	n.a.
**Total sexually transmitted pathogens**	**175**	**100**	**38**	**100**	**22**	**100**	**174**	**100**						
**Total ST pathogens/total tested population (*n* = 1047),%**	**16.71**		**3.63**		**2.10**		**16.62**							
*Streptococcus beta-haemolyticus* Gr. B	55	35.71	14	31.82	11	39.29	63	37.72	0.73	0.81	0.79	0.65	0.61	0.91
*Haemophylus parainfluenzae*	32	20.78	5	11.36	5	17.86	38	22.75	0.23	0.99	0.73	0.51	0.21	0.81
*Staphylococcus* spp. (coagulase-negative)	28	18.18	11	25.00	5	17.86	33	19.76	0.41	0.99	0.76	0.77	0.54	0.99
*Corynebacterium seminale*	12	7.79	4	9.09	4	14.29	6	3.59	0.76	0.29	0.12	0.71	0.22	0.052
*Streptococcus agalactiae*	9	5.84	9	20.45	2	7.14	3	1.80	**0.018**	0.68	0.081	0.31	**0.0001**	0.16
*Gardnerella Vaginalis*	5	3.25	0	0.00	0	0.00	6	3.59	n.a.	n.a.	0.99	n.a.	n.a.	n.a.
*Serratia mascescens*	4	2.60	0	0.00	0	0.00	4	2.40	n.a.	n.a.	0.99	n.a.	n.a.	n.a.
*Peptostreptococcus* spp. (AC)	3	1.95	1	2.27	0	0.00	3	1.80	0.99	n.a.	0.99	n.a.	0.99	n.a.
*Kocuria* spp.	2	1.30	0	0.00	1	3.57	6	3.59	n.a.	0.40	0.28	n.a.	n.a.	0.99
*Actinomyces* spp.	2	1.30	0	0.00	0	0.00	2	1.20	n.a.	n.a.	0.99	n.a.	n.a.	n.a.
*Clostridium* spp.	1	0.65	0	0.00	0	0.00	1	0.60	n.a.	n.a.	0.99	n.a.	n.a.	n.a.
*Acinetobacter* spp.	1	0.65	0	0.00	0	0.00	2	1.20	n.a.	n.a.	0.99	n.a.	n.a.	n.a.
**Total other pathogens**	**154**	**100**	**44**	**100**	**28**	**100**	**167**	**100**						
**Total other pathogens/total tested population (*n* = 1047),%**	**14.71**		**4.20**		**2.67**		**15.95**							
**Total pathogens detected, *n***	**872**		**278**		**165**		**794**							
**Total pathogens detected/total tested population (*n* = 1047),%**	**83.29**		**26.55**		**15.76**		**75.84**		**<0.0001**	**<0.0001**	0.15	**<0.0001**	**<0.0001**	**<0.0001**
**% Traditional uropathogens/total pathogens detected**	**62.27**		**70.50**		**69.70**		**57.05**		0.25	0.39	0.27	0.94	0.054	0.13
**% ST pathogens/total pathogens detected**	**20.07**		**13.67**		**13.33**		**21.91**		**0.044**	0.088	0.45	0.93	**0.013**	**0.038**
**% other pathogens/total pathogens detected**	**17.66**		**15.83**		**16.97**		**21.03**		0.55	0.85	0.15	0.78	0.12	0.33
**Total%**	**100**		**100**		**100**		**100**							

5G, five-glass test; 4G, four-glass test; 2G, two-glass test; VB2-S, VB2-semen test; n.a., not assessable (odds ratio = 0 or =∞); ST, sexually transmitted; AC, anaerobic conditions.

## Data Availability

Data supporting the reported results can be requested from the corresponding author. Data regarding single patients cannot be disclosed for reasons of privacy and confidentiality.

## Abbreviations

The following abbreviations are used in this manuscript:

CP	Chronic prostatitis
CP/CPPS	Chronic prostatitis/chronic pelvic pain syndrome
NIH-CPSI	NIH-Chronic Prostatitis Symptom Index
PPMT	Pre- and post-massage test
IPSS	International Prostate Symptom Score
PSA	Prostate-specific antigen
EPS	Expressed prostatic secretion
